# Development and validation of a prognostic model for predicting 30-day mortality risk in medical patients in emergency department (ED)

**DOI:** 10.1038/srep46474

**Published:** 2017-04-12

**Authors:** Duc T. Ha, Tam Q. Dang, Ngoc V. Tran, Thao N. T. Pham, Nguyen D. Nguyen, Tuan V. Nguyen

**Affiliations:** 1 Intensive Care Unit, National Hospital of Can Tho, Vietnam; 2 Research Center for Genetics and Reproductive Health, School of Medicine, Vietnam National University, Ho Chi Minh City, Vietnam; 3 Van Phuoc Mekong Hospital, Can Tho, Vietnam; 4 Department of Internal Medicine, University of Medicine and Pharmacy in Ho Chi Minh City, Vietnam; 5 Department of Intensive Care Medicine, Emergency Medicine and Clinical Toxicology, University of Medicine and Pharmacy in Ho Chi Minh City, Vietnam; 6 Intensive Care Unit, Cho Ray Hospital, Ho Chi Minh City, Vietnam; 7 Ton Duc Thang University, Ho Chi Minh City, Vietnam; 8 Garvan Institute of Medical Research, Sydney, Australia; 9 School of Public Health and Community Medicine, University of New South Wales, Sydney, Australia; 10 Centre for Health Technologies, University of Technology, Sydney, Australia

## Abstract

The primary aim of this prospective study is to develop and validate a new prognostic model for predicting the risk of mortality in Emergency Department (ED) patients. The study involved 1765 patients in the development cohort and 1728 in the validation cohort. The main outcome was mortality up to 30 days after admission. Potential risk factors included clinical characteristics, vital signs, and routine haematological and biochemistry tests. The Bayesian Model Averaging method within the Cox’s regression model was used to identify independent risk factors for mortality. In the development cohort, the incidence of 30-day mortality was 9.8%, and the following factors were associated with a greater risk of mortality: male gender, increased respiratory rate and serum urea, decreased peripheral oxygen saturation and serum albumin, lower Glasgow Coma Score, and admission to intensive care unit. The area under the receiver operating characteristic curve for the model with the listed factors was 0.871 (95% CI, 0.844–0.898) in the development cohort and 0.783 (95% CI, 0.743–0.823) in the validation cohort. Calibration analysis found a close agreement between predicted and observed mortality risk. We conclude that the risk of mortality among ED patients could be accurately predicted by using common clinical signs and biochemical tests.

Medical patients admitted to Emergency Department (ED) are highly heterogeneous in terms of disease spectrum and severity. Mortality is the most important outcome of ED care, and the rate of mortality can be used as a means for better prioritization of care and resource allocation. The rate of mortality among ED patients is high^1^. In ED, the death of a patient is commonly preceded by a cumulative deterioration of vital signs and clinical abnormalities^2^,^3^. Therefore, several prognostic models, including the Rapid Emergency Medicine Score^4^, Rapid Acute Physiology Score^5^ and Worthing Physiological Scoring system^6^, have been developed to make use of the clinical signs and abnormalities for predicting the risk of death in ED patients.

In a previous study^7^, we demonstrated that the above algorithms had good prognostic performance in the prediction of 30-day mortality in a tertiary hospital in Vietnam. However, the degree of discrimination, as measured by the area under the receiver operating characteristic curve (AUC), was between 0.70 and 0.80^7^, suggesting that there is room for further improvement of the existing prognostic models. In developing countries, EDs are normally overcrowded, and staff constantly struggle with an overwhelming number of patients from unplanned admissions. Moreover, the condition of care and disease severity among patients in developing countries are different from those in industrialized countries, and the difference calls for new prognostic models that can be applicable to ED patients in developing countries.

The present study sought to develop and validate a clinical predictive model for predicting 30-day mortality risk in ED patients by using routinely collected clinical, physiological and vital signs. We demonstrated that there exists a series of models that have comparable predictive accuracy, and that these models can be used to identify high-risk patients in ED. The models reported here can empower medical care providers to individualise ED care and optimise ED utilisation.

## Results

The study was carried out in two cohorts: development and validation. The two cohorts were recruited from two independent hospitals. The development cohort was used to derive predictive models, and the validation cohort was used for testing the predictive models.

Between 13 March 2013 and 1 June 2013, we had enrolled 2175 medical patients in ED for development cohort. However, after excluding patients who did not meet the inclusion criteria and patients who withdrew from the study, 1765 patients remained for the model development. During the follow-up period, the 30-day incidence of mortality in the development cohort was 9.8% (n = 173). Baseline characteristics of patients in the development cohort stratified by mortality status are shown in Table 1. The average age of participants was 65.8 years (range: 16–105 years). There was no statistically significant difference in age between survivors and deceased.

Bivariate analysis (Table 2) showed that the following risk factors were associated with an increased risk of mortality: male gender, advancing age, increased pulse, increased body temperature, lower systolic blood pressure and diastolic blood pressure, increased respiratory rate, reduced peripheral oxygen saturation, increased duration of illness, and lower Glasgow Coma Score (GCS). Moreover, cardiopulmonary resuscitation, mechanical ventilation, and admission to intensive care unit (ICU) were each associated with increased risk of mortality. Among comorbidities, cancer, chronic renal failure, cirrhosis, chronic respiratory failure, heart failure, and diabetes mellitus were also associated with a greater risk of mortality in ED patients.

### Model development

The Bayesian Model Average (BMA) algorithm identified 3 most parsimonious models for predicting the risk of 30-day mortality. The three models included the following factors: gender, respiratory rate, peripheral oxygen saturation, duration of illness, GCS, ICU admission, serum urea, glycaemia, serum albumin, alanine aminotransferase (ALT), and high-sensitivity C-reactive protein (hsCRP).

Model I included 10 factors and had a posterior probability of 5.5%; model II included 8 factors with 4% posterior probability; and model III included 11 factors with 3.8% posterior probability. Several risk factors were present in different models. The common factors in all three models were: gender, respiratory rate, peripheral oxygen saturation, duration of illness, GCS, ICU admission, serum urea, and serum albumin. The hazard ratio (HR) and 95% CI associated with each factor and each model are shown in Table 3. Among the risk factors, ICU admission was associated with the greatest risk or mortality, with the average HR ranging from 5.6 (Model I) to 6.6 (Model II).

**Figure 1:**
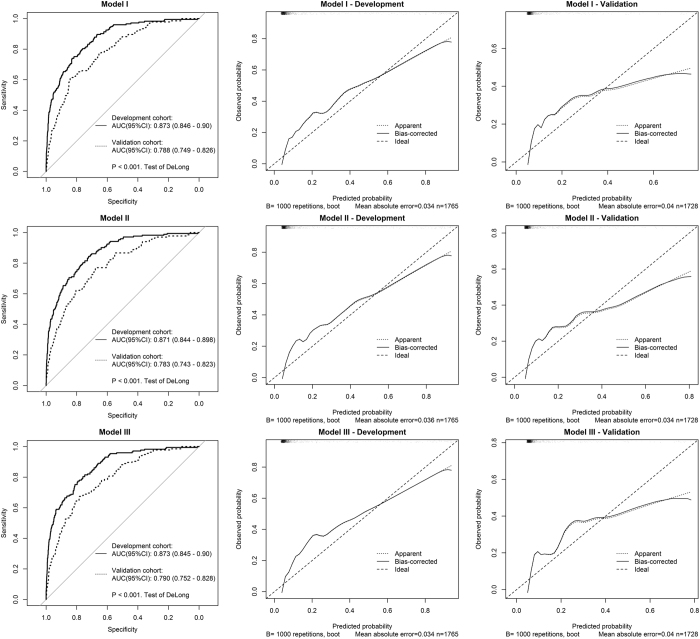
Area under the receiver operating characteristic curve (left panel) and calibration plot of three parsimonious models (middle and right panels).

The AUC for the three models (Fig. 1) were comparable. Model I yielded the AUC value of 0.873 (95% CI, 0.848–0.90), not significantly different from that of Model II (AUC = 0.871; 95% CI, 0.844–0.898) or Model III (AUC = 0.873; 95% CI, 0.845–0.90). From these results, it appeared that Model II was the most optimal model, because it has the least number of risk factors but yielded good discriminatory power.

### Model validation

**Figure 2:**
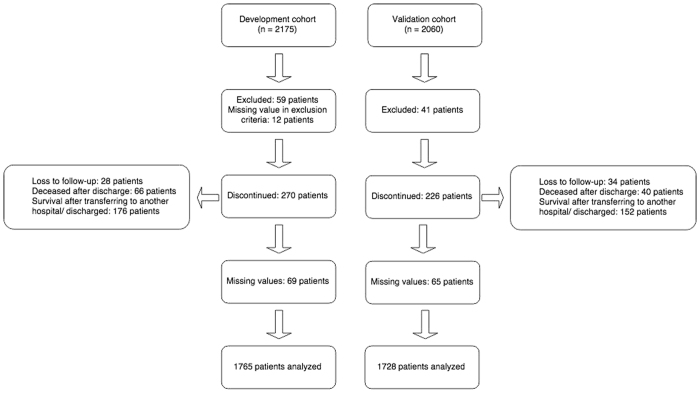
CONSORT diagram: Description of recruitment of study participants for the development and validation cohort.

**Figure 3:**
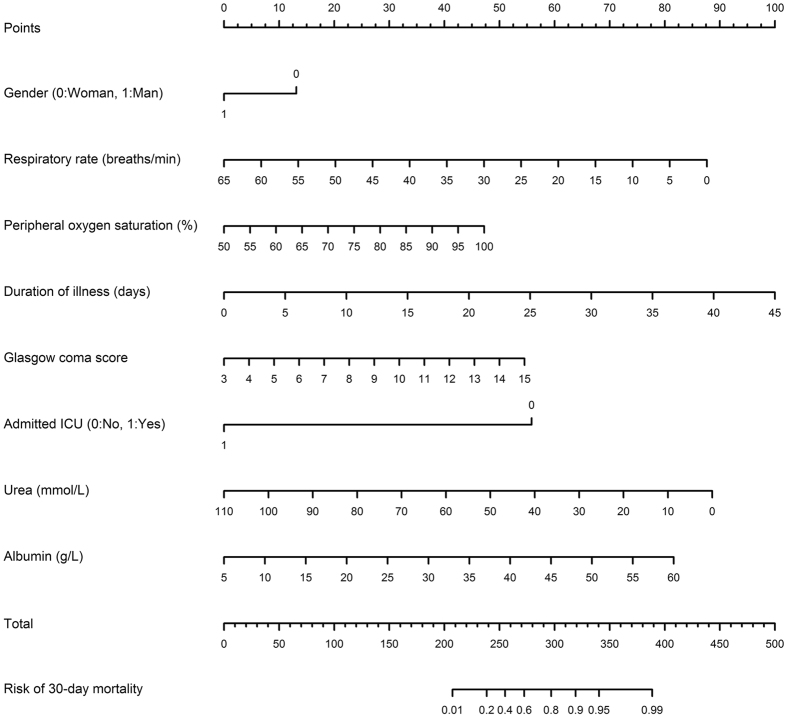
Nomogram for predicting the 30-day mortality risk based on Model II. *Instruction for usage*: Mark the gender of an individual on the “Gender” axis and draw a vertical line to the “Points” axis to determine how many points toward the probability of mortality the individual receives for his/her gender value. Repeat the process for each additional predictor. Sum the points of the predictors. Locate the final sum on the “Total” axis. Draw a vertical line down to the 30-day risk line to find the individual’s probability of sustaining mortality within 30 days (ICU: Intensive care unit).

Between 19 October 2013 and 31 March 2014, 2060 patients had been recruited for the validation cohort. After excluding patients who did not meet the inclusion/exclusion criteria, 1728 patients were available for analysis (Fig. 2). The incidence of 30-day mortality was 7.8% (n = 135), and this incidence rate was slightly lower than that in the development cohort (*P = *0.04, Fisher’s exact test). When the models derived from the development cohort were applied to predict the risk of mortality in the validation cohort, we found that there was a good agreement between observed and predicted risk of death. The maximum calibration error in predicting probability of mortality was about 4% for Model I and Model III, and 3.4% for Model II. In general, the AUC values obtained from the validation cohort was slightly lower than those obtained from the development cohort, but all AUC values were close to 0.8, a level deemed to be good discrimination. For example, the AUC for Model I, II and III was 0.788, 0.783, and 0.790, respectively (Fig. 1). Based on parameter estimates of Model II, a nomogram was built for individualising the risk of 30-day mortality (Fig. 3).

## Discussion

Emergency Departments worldwide are typically overcrowded and understaffed, and these problems have recently become a topic of discussion in emergency medicine. In developing countries, the problem is even more severe as EDs are often under-resourced. Under these circumstances, a risk stratification-based prioritization of care is a sensible approach. However, predicting the risk of mortality in EDs is a challenging task, because patients are not only highly heterogeneous but also medically complicated. In this study, by using a Bayesian approach to combine common clinical and biochemistry tests, we have developed and validated a series of predictive models for individualising the risk of 30-day mortality in ED patients. These models have proven good discrimination and calibration in external validation.

It is commonly thought that clinician’s assessment or clinical intuition can accurately predict the risk of mortality in EDs. However, when dealing with multiple risk factors, clinician’s assessment can be problematic, because they are unable to weigh information in a rational and objective manner. The lack of objectivity can result in inconsistent risk assessment between clinicians^8^. In some cases, considerable discrepancies between clinical intuition and actual mortality^9^,^10^. Therefore, in the presence of multiple risk factors, some of which may be potentially important, statistical thinking offers clinicians some useful guidance for dealing with uncertainty of risk assessment.

One of the most important aspects of statistical model building is to find relevant risk factors that have good predictive values. Typically, researchers develop a hypothesis about the relationship between risk factors and outcome, and then apply an algorithm, mostly stepwise regression, to identify a set of possible relevant risk factors. However, this classical approach suffers from two shortcomings that the stepwise method tends to identify redundant variables^11^, and that it produces only one “final” model. In reality, there are more than one competing model that can explain the relationship. A better approach is Bayesian Model Averaging which was introduced to scientific research about 20 years ago^12^, but has not been widely used in medical research. The Bayesian Model Averaging method has been shown to have superior and more robust performance than the stepwise method in the identification of relevant risk factors^11^,^13^. In this study, by using the Bayesian Model Averaging approach we have identify three sets of risk factors that can help clinicians estimate the risk of mortality in EDs patients.

Some risk factors for mortality identified in the present study have also been identified in previous studies^6^,^14^,^15^,^16^,^17^. It is well known that in ED men have a greater risk of mortality than women^18^, and this was also observed in our study. In clinical setting, about 45% of patients who died had antecedent abnormal vital signs, including respiratory distress, from 8 hours to 48 hours^19^. Moreover, among patients with normal vital signs at admission, 30% will be deteriorated within 24 hours^20^.

Tachypnea was a predictor of mortality in this study after adjusting for other predictors, and this finding is consistent with previous studies^4^,^5^,^6^,^16^. Peripheral oxygen saturation, another index for monitoring respiration, is also a known risk factor for mortality^4^,^6^,^16^,^17^. Low oxygen saturation (<90%) increases the odds of mortality within 24 hours by five-fold^17^, and in our study, decrease in peripheral oxygen saturation had a less pronounced effect, with a hazard ratio of 1.17 after adjusting for other covariates in the model.

In the present study, we found that decreased GCS, increased serum urea, increased glycaemia, decreased serum albumin, increased ALT, and increased hsCRP were independently associated with increased mortality risk. These associations have also been reported by various studies^15^,^21^,^22^,^23^,^24^,^25^. Interestingly, we found that a decision of ICU admission was strongly associated with mortality. Indeed, the risk of mortality among patients with this indication was increased by 6.6-fold compared with those without the indication. It could be argued that decision of ICU admission reflects a “clinical impression” which is known to be a good predictor of mortality status^18^,^26^. This finding suggests that clinical intuition can be an important factor, in addition to other measured factors, in the assessment of mortality in ED patients.

A number of multivariable models for predicting mortality in ED patients have been developed, and in validation cohorts, these models have good discrimination. For instance, the AUC for the Early Warning Score (7 factors), Simple Clinical Score (14 factors), Rapid Emergency Medicine Score (7 factors), and Worthing Physiological Scoring system (5 factors) for predicting mortality was 0.73^27^, 0.826^28^, 0.712^7^, and 0.797^7^, respectively. Among the models, only the Rapid Emergency Medicine Score (REMS) and Worthing Physiological System (WPS) were externally validated in developing countries. However, none of the available prognostic models appears to be suitable for individualised prognosis in ED patients. Our models have comparable AUC to those existing models. In previous studies, combining physician’s prediction with an objective model did not substantially improve the discrimination of mortality^26^; however, in our study, the integration of physician’s decision of ICU admission with objective models markedly increased the discrimination.

Our results should be interpreted within context of strengths and potential limitations. The study was designed as a prospective investigation with large sample size which allows us to define modest associations between risk factors and mortality. We have rigorously validated our models in a totally independent cohort which avoided the problem of over-fitting commonly found in previous studies. Moreover, we used sophisticated statistical approaches to derive at a series of predictive models, not just a single model that was reported in previous studies. In our view, there exists more than one model that can help predict mortality status, and the reliance on a single model is an underestimate of the uncertainty in the data. However, since patients’ information were collected at one time point, which might not reflect a full spectrum of a patient’s dynamics. The decision of ICU admission is, of course, not objective, and could potentially be a bias.

In summary, we have developed and validated new prognostic models to predict the risk of 30-day mortality in medical ED patients. The models will allow accurate risk assessment to identify high risk patients, and help optimize resources and patient management in ED.

## Methods

### Setting and patients

The study was conducted at the Can Tho National Hospital, Vietnam (for development cohort) and the Can Tho General Hospital, Vietnam (for validation cohort). The Can Tho National Hospital is a tertiary teaching hospital that serves 17 million residents in the Mekong Delta region; its ED on average admits 75 non-surgical patients per day. The Can Tho General Hospital has 400 beds, providing care to the residents of Can Tho City; on average, the ED admits 50 non-surgical patients per day.

We enrolled medical patients (non-trauma and non-surgical) from the two emergency departments between 13 March 2013 and 31 March 2014. The inclusion criteria were: all patients aged 16 years and older and who could give informed consent. Patients were excluded from the study if they had one of the following conditions: acute coronary syndrome, burns, cardiac arrest before admitting to the hospital or which occurred in the ED with failure of cardiopulmonary resuscitation, snakebite, insect bite or sting, poisoning (drugs, alcohol, intoxication, paraquat, insecticides, rodenticides, corrosive substances). We also excluded patients with burns, cardiac arrest with failure of cardiopulmonary resuscitation. Women in labor and dead-on-arrival patients were also excluded from the study. The study protocol and procedure were approved by the Can Tho National Hospital ethics committee. All patients gave written informed consent. Patients could withdraw from the study at any time without giving reasons. All methods were performed in accordance with the relevant guidelines and regulations.

### Study procedure

All patients who met inclusion and exclusion criteria were invited to participate in the study. Upon giving the written informed consent, data collection was conducted by a trained research worker by using a structured questionnaire. The questionnaire collected data concerning demographic characteristics, medical history, physiological data, haematological and biochemistry tests (see Supplementary Table S1). After 30 days of admission, the research worker would contact patients or relatives or guardians to obtain information on survival status. Patients who had stayed in hospital for more than 30 days were considered “censored”.

### Outcome measure

The primary outcome of the study was mortality occurred within 30 days of hospital admission. Mortality was defined as (1) death in hospital from any cause; (2) family-initiated discharge and death either on the way home or within 24 hours after discharge; (3) doctor-initiated discharge and death at home. It should be noted that in Vietnamese culture, when a patient was in the end stage of disease, the patient or family often requests for discharge from hospital because they prefer to pass away at home.

### Risk factors

Risk factors considered in this study included vital signs, peripheral oxygen saturation, duration of illness, GCS (see Supplementary Table S2), cardiopulmonary resuscitation, mechanical ventilation, ICU admission, functional status, comorbidity, haematological and biochemical test (see Supplementary Table S1). Blood pressure and pulse rate were measured electronically (OMRON HEALTHCARE Co, Vietnam) and rechecked manually where blood pressure was either too low or too high. Peripheral oxygen saturation was measured by an electronic device (NONIN Co, USA). Decision of ICU admission was made by a senior attending physician after considering laboratory tests and primary diagnosis.

### Data management and analysis

Data were entered into a designed database twice. The first data entry was made within a day after admission. The second data entry was undertaken at the termination of the study. Data from the two entries were used to check for potential inconsistencies, and any inconsistency was adjudicated with the original patient record.

Data analysis was performed according to a statistical analysis plan prior to the collection of data. First, we used descriptive statistics (i.e., mean, standard deviation, median with interquartile range, proportion) for each clinical and laboratory variable with stratification by survival status. Difference between groups in categorical variables was tested by the Fisher’s exact test. The normal distribution of continuous variables was tested by the Shapiro-Wilk normality test. The association between continuous variables and 30-day mortality was tested by the Student’s *t* test for normally distributed variables or Mann-Whitney test for non-normally distributed variables.

Second, we used the Cox’s proportional hazards model^29^ to assess the association between potential risk factors and 30-day mortality (i.e. hazard ratio) from the development cohort. Since there were several potential risk factors and the number of “potential models” for predicting 30-day mortality can be very large, the Bayesian Model Averaging (BMA)^30^ was used to search for the most parsimonious models from the development cohort. This approach has been shown to have superior performance compared to “traditional” approaches such as stepwise regression^11^,^13^. In the BMA approach, the regression analysis was performed for 2^M^ (where M is the number of risk factors) competing models. The BMA averaged point estimates for regression coefficient over the all possible models. BMA produces a posterior probability of each possible model and posterior probability for regression coefficient associated with each genetic variant. The posterior probability is a function of a prior probability and the likelihood of a model. In this study, given the large number of genetic variant and there is little information available for eliciting prior distributions, we used the “uninformative” prior distributions, that a priori, make all models and parameters equally likely are appealing. Thus, in BMA, we consider a set of competing models, not just a single model, to account for the variation in the data. The analysis was done with the R statistical environment^31^ and the BMA package^32^.

The discrimination of the most parsimonious models was estimated by the AUC^33^ for the development and validation cohort. The difference between two AUCs was tested by the Delong method^34^. Agreement between observed mortality and predicted mortality was evaluated using the bootstrap technique. In this technique, 1000 sub-samples, each with 500 subjects, of the entire sample were repeatedly sampled (with replacement) and analyzed^35^. Based on the parameters selected from the most parsimonious models, a nomogram using the “rms” library^35^,^36^ was constructed for predicting 30-day mortality. In a further validation, the predicted probability of 30-day mortality was compared with the observed probability on the development cohort and validation cohort. All statistical analyses were performed with the R Statistical Environment version 3.1.0 on Windows platform^37^.

## Additional Information

**How to cite this article:** Ha, D. T. *et al*. Development and validation of a prognostic model for predicting 30-day mortality risk in medical patients in emergency department (ED). *Sci. Rep.* **7**, 46474; doi: 10.1038/srep46474 (2017).

**Publisher's note:** Springer Nature remains neutral with regard to jurisdictional claims in published maps and institutional affiliations.

## Supplementary Material

Supplementary Information

## Figures and Tables

**Table 1:** Clinical characteristics of 1765 patients in the development cohort and 1728 patients in the validation cohort classified by mortality status.

Characteristics	Development cohort		Validation cohort	
	Survivors	Deceased	Survivors	Deceased
Number of patients	1592	173	1593	135
Number of man (%)	716 (45.0)	98 (56.6)*	704 (44.2)	65 (48.1)
Age (years)	68 [55, 80]	71 [59, 81]	64 [51, 77]	74 [60, 83]**
Pulse (per min)	90 [79, 103]	100 [83, 115]**	87 [78, 101]	100 [85, 112]**
Body temperature (oC)	37.1 [37.0, 37.7]	37.3 [37.0, 38.1]*	37.0 [37.0, 37.5]	37.0 [37.0, 37.5]
Systolic blood pressure (mmHg)	139 [120, 159]	131 [110, 158]*	135 [120, 150]	130 [104, 150]**
Diastolic blood pressure (mmHg)	80 [70, 90]	78 [63, 90]	80 [70, 90]	75 [61, 88]*
Respiratory rate (breaths/min)	22 [20, 26]	26 [22, 30]**	22 [20, 24]	24 [20, 26]**
Peripheral oxygen saturation (%)	97 [94, 99]	94 [87, 97]**	99 [97, 99]	96 [90, 99]**
Duration of illness (days)	1 [0, 3]	1 [0, 2]	0 [0, 2]	0 [0, 2]
Glasgow Coma Score	15 [15, 15]	15 [10, 15]**	15 [15, 15]	15 [15, 15]**
Cardiopulmonary resuscitation	1 (0.1)	3 (1.7)*	0 (0.0)	2 (1.5)*
Mechanical ventilation (n; %)	14 (0.9)	25 (14.5)**	5 (0.3)	8 (5.9)**
Admitted intensive care unit (n; %)	32 (2.0)	63 (36.4)**	40 (2.5)	31 (23.0)**
Functional status				
Independent (n; %)	1373 (86.2)	138 (79.8)*	1346 (84.5)	97 (71.9)**
Partially dependent (n; %)	164 (10.3)	21 (12.1)	184 (11.6)	23 (17.0)
Completely dependent (n; %)	55 (3.5)	14 (8.1)	63 (4.0)	15 (11.1)
Length of stay (days)	7 [4, 10]	3 [1, 8]**	5 [3, 7]	5 [2, 10]
Immunocompromised by agent (n; %)	28 (1.8)	4 (2.3)	5 (0.3)	1 (0.7)
Lymphoma (n; %)	2 (0.1)	1 (0.6)	0 (0.0)	0 (0.0)
Leukemia or myeloma (n; %)	8 (0.5)	3 (1.7)	0 (0.0)	1 (0.7)
Cancer (n; %)	18 (1.1)	5 (2.9)	9 (0.6)	3 (2.2)
Chronic renal failure (n; %)	67 (4.2)	10 (5.8)	56 (3.5)	11 (8.1)*
Chronic respiratory failure (n; %)	88 (5.5)	19 (11.0)*	135 (8.5)	19 (14.1)*
Cirrhosis with ascites (n; %)	44 (2.8)	10 (5.8)*	38 (2.4)	7 (5.2)
Heart failure (n; %)	87 (5.5)	24 (13.9)**	272 (17.1)	35 (25.9)*
Diabetes mellitus (n; %)	539 (33.9)	75 (43.4)*	206 (12.9)	16 (11.9)
Haemoglobin (g/dL)	12.3 [10.7, 13.6]	12.0 [9.9, 13.4]*	—	—
Leukocyte (x 103/μL)	9.2 [7.2, 12.4]	11.7 [8.5, 16.0]**	—	—
Platelet (x 103/ μL)	222 [171, 277]	203 [117, 258]**	—	—
Serum urea (mmol/L)	5.7 [4.2, 8.2]	7.9 [5.0, 13.7]**	5.1 [3.7, 7.3]	7.2 [4.6, 11.7]**
Glycaemia (mmol/L)	6.2 [5.3, 7.9]	7.4 [5.8, 10.2]**	6.5 [5.5, 8.3]	7.1 [5.9, 9.3]*
Serum creatinine (μmol/L)	94 [79, 118]	112 [91, 158]**	100 [84, 122]	123 [89, 170]**
Serum albumin (g/L)	38 [34, 42]	34 [29, 40]**	40 [36, 44]	35 [28, 39]**
AST (UI/L)	27 [21, 41]	42 [26, 90]**	—	—
ALT (UI/L)	21 [14, 35]	26 [17, 47]**	19 [13, 30]	23 [14, 45]*
hsCRP (mg/dL)	0.5 [0.1, 3.0]	1.5 [0.4, 7.9]**	0.6 [0.2, 2.6]	2.6 [0.7, 9.5]**
A1c (%)	5.9 [5.4, 6.7]	6.3 [5.6, 7.2]**	—	—

*P-value < 0.05. **P-value ≤ 0.001.

AST, aspartate aminotransferase. ALT, alanine aminotransferase. hsCRP, high-sensitivity C-reactive protein. A1c, glycated haemoglobin.

**Table 2:** Risk factors for 30-day mortality from the development cohort: bivariate analysis.

Risk factor	Unit	Hazard ratio (95% CI)	P-value
Gender	Man	1.56 (1.15–2.10)	0.004
Age (years)	+10	1.06 (0.97–1.16)	0.188
Pulse (per min)	+10	1.17 (1.10–1.24)	<0.001
Body temperature (°C)	+1	1.38 (1.18–1.61)	<0.001
Systolic blood pressure (mmHg)	−5	1.03 (1.0–1.05)	0.043
Diastolic blood pressure (mmHg)	−5	1.03 (0.99–1.08)	0.135
Respiratory rate (breaths/min)	+5	1.44 (1.32–1.57)	<0.001
Peripheral oxygen saturation (%)	−5	1.57 (1.47–1.68)	<0.001
Duration of illness (days)	+5	0.78 (0.60–1.0)	0.053
Glasgow Coma Score	−1	1.35 (1.30–1.40)	<0.001
Cardiopulmonary resuscitation	Yes	16.63 (5.3–52.19)	<0.001
Mechanical ventilation	Yes	13.22 (8.63–20.26)	<0.001
Admitted intensive care unit	Yes	17.90 (13.09–24.49)	<0.001
Functional status			
Independent	Yes	1.0 (reference)	
Partially dependent	Yes	1.24 (0.78–1.97)	0.355
Completely dependent	Yes	2.38 (1.37–4.12)	0.002
Immunocompromised by agent	Yes	1.29 (0.48–3.47)	0.617
Lymphoma	Yes	3.55 (0.50–25.36)	0.206
Leukemia or myeloma	Yes	2.92 (0.93–9.16)	0.065
Cancer	Yes	2.48 (1.02–6.03)	0.046
Chronic renal failure	Yes	1.34 (0.71–2.53)	0.371
Chronic respiratory failure	Yes	2.01 (1.25–3.24)	0.004
Cirrhosis with ascites	Yes	2.02 (1.07–3.83)	0.031
Heart failure	Yes	2.55 (1.66–3.92)	<0.001
Diabetes mellitus	Yes	1.47 (1.09–1.99)	0.012
Haemoglobin (g/dL)	−1	1.06 (1.0–1.12)	0.049
Leukocyte (x 10^3^/μL)	+1	1.01 (1.0–1.02)	<0.001
Platelet (x 10^3^/ μL)	−10	1.03 (1.02–1.05)	<0.001
Serum urea (mmol/L)	+5	1.16 (1.10–1.21)	<0.001
Glycaemia (mmol/L)	+5	1.30 (1.20–1.42)	<0.001
Serum creatinine (μmol/L)	+20	1.01 (1.01–1.02)	0.003
Serum albumin (g/L)	−5	1.34 (1.22–1.48)	<0.001
AST (UI/L)	+20	1.008 (1.005–1.012)	<0.001
ALT (UI/L)	+20	1.02 (1.02–1.03)	<0.001
hsCRP (mg/dL)	+5	1.37 (1.25–1.49)	<0.001
A1c (%)	+1	1.07 (1.0–1.15)	0.058

AST, aspartate aminotransferase. ALT, alanine aminotransferase. hsCRP, high-sensitivity C-reactive protein. A1c, glycated haemoglobin.

**Table 3:** Association between risk factors and 30-day mortality risk: results of multivariable analyses.

Model	Unit	Hazard ratio (95% CI)	P-value
**Model I**			
Gender	Man	1.61 (1.18–2.20)	0.003
Respiratory rate (breaths/min)	+5	1.24 (1.12–1.37)	<0.001
Peripheral oxygen saturation (%)	−5	1.17 (1.07–1.27)	<0.001
Duration of illness (days)	+5	0.67 (0.51–0.88)	0.004
Glasgow Coma Score	−1	1.19 (1.13–1.25)	<0.001
Admitted intensive care unit	Yes	5.57 (3.73–8.31)	<0.001
Serum urea (mmol/L)	+5	1.12 (1.05–1.20)	0.001
Glycaemia (mmol/L)	+5	1.19 (1.05–1.35)	0.005
Serum albumin (g/L)	−5	1.31 (1.18–1.47)	<0.001
ALT (U/L)	+20	1.02 (1.01–1.03)	<0.002
**Model II**			
Gender	Man	1.53 (1.13–2.09)	0.007
Respiratory rate (breaths/min)	+5	1.25 (1.13–1.38)	<0.001
Peripheral oxygen saturation (%)	−5	1.17 (1.08–1.28)	<0.001
Duration of illness (days)	+5	0.67 (0.51–0.87)	0.003
Glasgow Coma Score	−1	1.18 (1.12–1.24)	<0.001
Admitted intensive care unit	Yes	6.59 (4.45–9.74)	<0.001
Serum urea (mmol/L)	+5	1.13 (1.06–1.20)	<0.001
Serum albumin (g/L)	−5	1.30 (1.17–1.45)	<0.001
**Model III**			
Gender	Man	1.59 (1.17–2.18)	0.003
Respiratory rate (breaths/min)	+5	1.21 (1.09–1.34)	<0.001
Peripheral oxygen saturation (%)	−5	1.17 (1.07–1.27)	<0.001
Duration of illness (days)	+5	0.66 (0.50–0.87)	0.003
Glasgow Coma Score	−1	1.19 (1.13–1.25)	<0.001
Admitted intensive care unit	Yes	5.60 (3.77–8.31)	<0.001
Serum urea	+5	1.11 (1.04–1.19)	0.003
Glycaemia	+5	1.18 (1.04–1.33)	0.008
Serum albumin	−5	1.26 (1.12–1.42)	<0.001
ALT	+20	1.02 (1.01–1.03)	<0.001
hsCRP	+5	1.12 (1.01–1.24)	0.029

ALT, alanine aminotransferase. hsCRP, high-sensitivity C-reactive protein.
