# Corrigendum: Development and validation of a prognostic model for predicting 30-day mortality risk in medical patients in emergency department (ED)

**DOI:** 10.1038/srep46919

**Published:** 2017-12-22

**Authors:** Duc T. Ha, Tam Q. Dang, Ngoc V. Tran, Thao N. T. Pham, Nguyen D. Nguyen, Tuan V. Nguyen

Scientific Reports 7: Article number: 46474; 10.1038/srep46474 published online: 04 12 2017; updated: 12 22 2017.

This Article contains errors in Figure 3 where the authors calculated the probability of survival instead of the probability of mortality. The correct Figure 3 appears below as Figure 1.

## Figures and Tables

**Figure 1:**
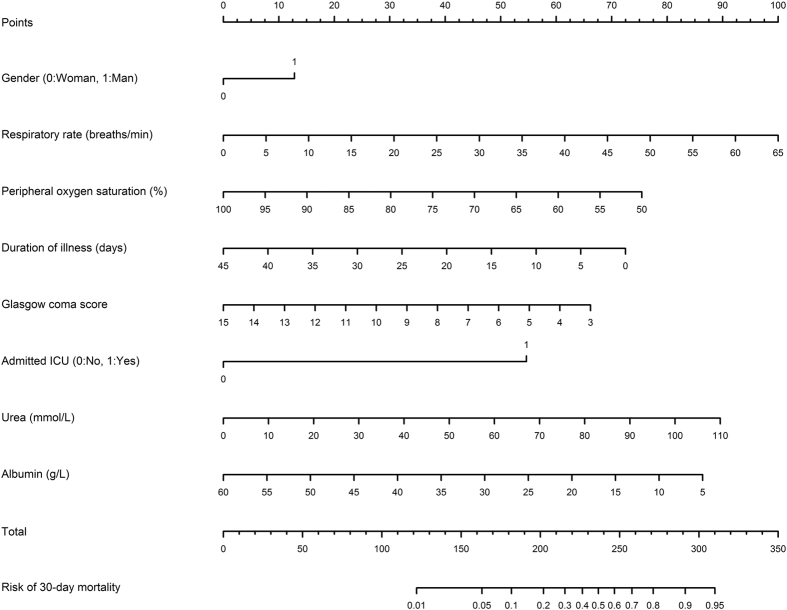
Spicule types and spicule formation in S. ciliatum.

