# Supplementary material for: Development and validation of a prognostic model for predicting 30-day mortality risk in medical patients in emergency department (ED)
Source: Sci Rep. 2017 Apr 12;7:46474. doi: 10.1038/srep46474 (PMC5388874; doi:10.1038/srep46474)
Supplement: Supplementary Information [file srep46474-s1.pdf]

Development and validation of a prognostic model for predicting 30-day mortality risk in medical patients in emergency department (ED)

*Duc T. Ha[1,2,3]
Email: hatanduc@gmail.com

Tam Q. Dang[1]
Email: tstamct@yahoo.com

Ngoc V. Tran[4]
Email: tranvanngocdhyd@yahoo.com

Thao N. T. Pham[5,6]
Email: thaocrh10@yahoo.com

Nguyen D. Nguyen[7]
Email: ngdinhnguyen@yahoo.com

Tuan V. Nguyen[7,8,9,10]
Email: t.nguyen@garvan.org.au

[1] Intensive Care Unit, National Hospital of Can Tho, Vietnam.
[2] Research Center for Genetics and Reproductive Health, School of Medicine, Vietnam National University, Ho Chi Minh City, Vietnam
[3] Van Phuoc Mekong Hospital, Can Tho, Vietnam.
[4] Department of Internal Medicine, University of Medicine and Pharmacy in Ho Chi Minh City, Vietnam.
[5] Department of Intensive Care Medicine, Emergency Medicine and Clinical Toxicology, University of Medicine and Pharmacy in Ho Chi Minh City, Vietnam.
[6] Intensive Care Unit, Cho Ray Hospital, Vietnam
[7] Ton Duc Thang University, Vietnam.
[8] Garvan Institute of Medical Research, Australia.
[9] School of Public Health and Community Medicine, University of New South Wales, Australia.
[10] Centre for Health Technologies, University of Technology, Sydney.

*** Corresponding author**
Dr. Duc Tan Ha
Intensive care unit
National Hospital of Can Tho
315. Nguyen Van Linh street, Ninh Kieu District, Can Tho city, Vietnam
Phone: +84 989216130
Email: hatanduc@gmail.com

Supplementary Information

Supplementary Table S1: Variables and method of measurement

Variable	Definition and method of measurement
Pulse	Enrolled within 15 minutes from admitting to Emergency Department (ED). Pulse was counted for a minute. If there was no pulse, pulse was recorded as zero. Locations of taking pulse were radial artery, brachial artery, carotid artery, femoral artery.
Body temperature	Maximal temperature was recorded at ED. Unit of measurement was Celsius degree. Body temperature was taken in axilla and plus 0.5 Celsius degree.
Blood pressure	Enrolled within 15 minutes from admitting to ED.
Respiratory rate	Enrolled within 15 minutes from admitting to ED. Respiratory rate was counted for a minute. If the patient was ventilated through tracheal tube, self-breathing rate was recorded. If the patient did not breathe within 15 seconds, breathing rate was recorded as zero.
Peripheral oxygen saturation	Enrolled within 15 minutes from admitting to ED. Peripheral oxygen saturation was checked for a minute with room air.
Duration of illness	The days that the patient's health was worse than baseline (not including the days of therapy from other hospital). The change from baseline could be sudden or gradual.
Glasgow coma score	Maximal Glasgow coma score which was recorded at ED.
Cardiopulmonary resuscitation	Cardiopulmonary resuscitation happened at ED or other hospital.
Mechanical ventilation	Invasive ventilation at ED.
Admitted intensive care unit	Decision of intensive care unit admission was made by a senior attending physician after considering laboratory tests and primary diagnosis
Functional status	The level of self-care confirmed by the physician on admission to ED, revealing the functional status of patient prior to admitting hospital [1].<br>- Totally dependent: the patient cannot complete any activities of daily living for himself or herself; includes patients who are totally dependent on nursing care (e.g. dependent nursing home patient).<br>- Partially dependent: the patient needs help from another person for some activities of daily living. Patients admitted from a nursing home setting who are not totally dependent would arrange into this category, as would any patient who requires hemodialysis or home ventilator support yet preserves some independent function.<br>- Independent: the patient is independent in activities of daily living; includes patient who needs usage of tool(s) or device(s) for independent activities of daily living.
Length of stay	Duration of hospitalization.
Immunocompromised by agent	Medical history of using cytotoxic agents within 3 months [2], or corticosteroid equivalent to 1mg/kg/day prednisone for greater than 1 month during 3 months preceding ED admission [3].
Lymphoma, leukemia, myeloma, and other cancer	History of diagnosis with lymphoma, leukemia, myeloma, or other cancer.
Chronic renal failure	History of indication of routine hemodialysis or peritoneal dialysis due to chronic renal failure.
Chronic respiratory failure	History of diagnosis with chronic respiratory disease and dyspnea with activities of daily living or at rest [4].

Supplementary Table S1: Variables and method of measurement

Variable	Definition and method of measurement
Cirrhosis with ascites	Evidence of ascites, prothrombin time less than 51%, and coarse hepatic parenchymal echotexture appearing on ultrasonography [5].
Heart failure	Heart failure was defined according to the criteria described in [6].
Diabetes mellitus	History of diagnosis with type 1 diabetes mellitus or type 2 diabetes mellitus, or hemoglobin A1c greater than 6.5% [7].
Hematological and biochemical tests	Enrolled within 24 hours from admitting to ED

Supplementary Table S2. **The Glasgow coma score (Teasdale and Jennett 1974, cited in [8])**

		Score
Eye opening	Spontaneous	4
	To speech	3
	To pain	2
	None	1
Best verbal response	Oriented	5
	Confused conversation	4
	Inappropriate words	3
	Incomprehensible sounds	2
	None	1
Best motor response	Obeys commands	6
	Localizes pain	5
	Withdrawal (normal flexion)	4
	Abnormal flexion (decorticate)	3
	Extension (decerebrate)	2
	None	1

References

1 Arozullah, A. M., Khuri, S. F., Henderson, W. G., Daley, J. & Participants in the National Veterans Affairs Surgical Quality Improvement, P. Development and validation of a multifactorial risk index for predicting postoperative pneumonia after major noncardiac surgery. *Annals of internal medicine* **135**, 847-857 (2001).

2 Fine, M. J. *et al.* Processes and outcomes of care for patients with community-acquired pneumonia: results from the Pneumonia Patient Outcomes Research Team (PORT) cohort study. *Archives of internal medicine* **159**, 970-980 (1999).

3 Nseir, S. *et al.* Relationship between immunosuppression and intensive care unit-acquired multidrug-resistant bacteria: a case-control study. *Critical care medicine* **35**, 1318-1323, doi:10.1097/01.CCM.0000261885.50604.20 (2007).

4 Arozullah, A. M., Daley, J., Henderson, W. G. & Khuri, S. F. Multifactorial risk index for predicting postoperative respiratory failure in men after major noncardiac surgery. The National Veterans Administration Surgical Quality Improvement Program. *Annals of surgery* **232**, 242-253 (2000).

5 Eric, G. & Sanjiv, C. (UpToDate, Inc, 2009).

6 Dickstein, K. *et al.* ESC Guidelines for the diagnosis and treatment of acute and chronic heart failure 2008: the Task Force for the Diagnosis and Treatment of Acute and Chronic Heart Failure 2008 of the European Society of Cardiology. Developed in collaboration with the Heart Failure Association of the ESC (HFA) and endorsed by the European Society of Intensive Care Medicine (ESICM). *European heart journal* **29**, 2388-2442, doi:10.1093/eurheartj/ehn309 (2008).

7 American Diabetes, A. Diagnosis and classification of diabetes mellitus. *Diabetes care* **32 Suppl 1**, S62-67, doi:10.2337/dc09-S062 (2009).

8 Sternbach, G. L. The Glasgow coma scale. *The Journal of emergency medicine* **19**, 67-71 (2000).
